# Risk Factors for Persistent Lymphedema Following Modified Axillary Lymph Node Dissection in Breast Cancer Patients: A Retrospective Single‐Center Study

**DOI:** 10.1155/ijbc/4135685

**Published:** 2026-09-28

**Authors:** Iraj Feizi, Bahareh Nikandam, Zohreh Shahabi, Amir Ahmad Arabzadeh

**Affiliations:** ^1^ Department of Surgery, School of Medicine, Ardabil University of Medical Sciences, Ardabil, Iran, arums.ac.ir; ^2^ Hematology, Oncology, and Stem Cell Transplantation Research Center, Research Institute for Oncology, Hematology, and Cell Therapy, Tehran University of Medical Sciences, Tehran, Iran, tums.ac.ir

**Keywords:** axillary lymph node dissection, breast cancer, chemotherapy, modified radical mastectomy, persistent lymphedema, risk factors

## Abstract

**Background:**

Persistent lymphedema (PLE) is a common complication following breast cancer surgery, particularly after axillary lymph node dissection (ALND). It significantly impacts the quality of life and daily functioning of survivors. Understanding the risk factors for PLE is crucial for early detection and effective management.

**Purpose:**

This study is aimed at identifying the risk factors for PLE following modified ALND in breast cancer patients, focusing on clinical, surgical, and molecular characteristics.

**Methods:**

A retrospective, single‐center study was conducted on women who underwent breast cancer surgery between 2018 and 2021. Demographic, clinical, and treatment‐related data were collected from medical records. Univariable and multivariable logistic regression analyses were performed to identify risk factors for PLE.

**Results:**

Of the 150 patients, 31 (21.4%) developed PLE. Significant risk factors for PLE included metastasis (*p* < 0.0001), neurovascular invasion (*p* = 0.029), and the presence of ER‐negative (*p* = 0.032) and Ki‐67‐positive (*p* = 0.007) tumors. Surgery type was significantly associated with PLE, with patients who underwent modified radical mastectomy (MRM) having a higher incidence (42.9%) compared with those who had breast‐conserving surgery (BCS) (16.0%) (*p* = 0.002). Chemotherapy also increased the likelihood of PLE (*p* = 0.001). Multivariable logistic regression revealed that metastasis was the strongest predictor of PLE (OR 11.91, 95% CI: 1.94–73.09, *p* = 0.007).

**Conclusions:**

Metastasis, surgery type, chemotherapy, and molecular factors such as ER and Ki‐67 expression showed unadjusted associations with PLE. However, in multivariable logistic regression, metastasis remained the only independent predictor of PLE. These findings support risk‐stratified postoperative surveillance, and larger prospective studies with standardized lymphedema measurement are needed to validate independent predictors.

## 1. Introduction

Advances in breast cancer treatment have significantly improved survival rates, resulting in an increasing population of long‐term survivors. Consequently, there is a growing need to address treatment‐related side effects to enhance the quality of life for these individuals. Among the most common complications following breast cancer surgery is breast cancer‐related lymphedema (BCRL), a condition that significantly impacts the physical and emotional well‐being of patients [1, 2].

Persistent lymphedema (PLE), a chronic form of BCRL, can severely impair daily functioning and quality of life [3]. PLE arises from dysfunction in the lymphatic system, leading to the accumulation of protein‐rich lymph fluid in the interstitial space, causing symptoms such as swelling, pain, and reduced limb mobility [4]. Because of the lack of standardized diagnostic criteria, the reported incidence of PLE varies widely, ranging from less than 5% to over 50% of breast cancer patients [5, 6]. The consequences of PLE are significant, contributing to both physical and psychological morbidity [7]. Some studies suggest that the presence of PLE may even be associated with reduced survival rates [8]. Although there is currently no cure for PLE, its symptoms can be managed, making early diagnosis and effective prevention strategies critical to mitigating its impact [9].

Research indicates that both clinical and treatment‐related variables significantly contribute to the development of PLE. Factors such as age, body mass index (BMI), the extent of surgery, radiation therapy, and the number of lymph nodes removed have been identified as key determinants in the onset and persistence of PLE [10–12]. Understanding these factors is crucial for early detection and management strategies that can mitigate the risk and severity of PLE in breast cancer patients.

Given the significant burden of PLE, identifying its risk factors—especially after modified axillary lymph node dissection (ALND)—is essential for improving prevention and management strategies. Although some studies have investigated risk factors for lymphedema after breast cancer surgery, few have specifically focused on modified ALND, which is one of the most commonly performed procedures. This study is aimed at filling this gap by identifying and evaluating the risk factors for PLE following ALND in breast cancer patients. By understanding these factors, we aim to develop more targeted prevention strategies and interventions, ultimately improving patient outcomes and quality of life.

## 2. Methods

### 2.1. Study Design and Setting

This retrospective, single‐center study included women who underwent breast cancer surgery between 2018 and 2021 at Imam Khomeini Hospital, affiliated with Ardabil University of Medical Sciences (ARUMS), Ardabil, Iran. The study protocol was approved by the Ethics Committee of ARUMS (IR.ARUMS.MEDICINE.REC.1400.008).

### 2.2. Participants

The inclusion criteria were women diagnosed with breast cancer who underwent surgery during the study period. Exclusion criteria were applied to reduce major competing causes of upper‐limb edema and extreme heterogeneity that could confound attribution to modified ALND. Specifically, we excluded severe obesity (BMI ≥ 35 kg/m^2^) (a strong independent predictor of lymphedema that may dominate risk estimates), postoperative infection (may cause inflammatory/transient swelling), significant trauma to the upper limbs or neck (alternative etiology for edema/lymphatic disruption), neoadjuvant radiotherapy and advanced stage (III–IV) (substantially different disease and treatment intensity and radiation fields that alter baseline lymphedema risk structure), and prior treatment at another institution (incomplete baseline and follow‐up documentation).

### 2.3. Data Collection

Demographic and clinical data were retrospectively collected from patients′ medical records to identify potential risk factors for PLE. The variables extracted included age, presence of PLE, tumor location, metastasis, cancer recurrence, tumor pathology, vascular and nerve invasion, estrogen (ER) and progesterone receptor (PR) status, human epidermal growth factor receptor 2 (HER2) expression, Ki‐67 expression, chemotherapy administration, number of dissected lymph nodes, lymph node positivity, and type of surgery performed.

### 2.4. Definition and ascertainment of PLE

PLE was defined as ipsilateral upper‐limb swelling consistent with BCRL that was documented during postoperative follow‐up and persisted for ≥ 3 months after onset (i.e., present on at least two follow‐up assessments separated by ≥ 3 months), without an alternative acute explanation (e.g., cellulitis or thrombosis). PLE ascertainment was based on standard clinical follow‐up documentation in medical records; when available, arm circumference measurements recorded in the chart were extracted, and when not available, physician‐documented persistent ipsilateral arm swelling consistent with lymphedema was used for classification.

### 2.5. Statistical Analysis

Data analysis was performed using SPSS software, Version 23. Descriptive statistics, including frequency distributions and means ± standard deviation (SD), were used to summarize the data. Independent *t*‐tests and chi‐square tests were applied to compare groups. Univariable logistic regression was performed for candidate predictors. Variables were considered for multivariable logistic regression based on clinical plausibility and univariable screening (*p* < 0.20), with emphasis on model parsimony given the limited number of PLE events. Because PLE occurred in 31 patients, we limited the number of covariates to reduce overfitting risk and report adjusted associations accordingly.

Analyses were conducted using a complete‐case approach for patients with sufficient documentation to determine PLE status and key covariates; no imputation was performed because missingness was primarily due to loss of evaluable follow‐up documentation (mortality/noncooperation) or incomplete records that prevented reliable outcome classification.

## 3. Results

### 3.1. Demographic and Clinical Characteristics

A total of 178 patients were screened; 28 were excluded due to mortality (*n* = 9), lack of cooperation/attendance for follow‐up assessment (*n* = 7), or incomplete records that prevented classification of PLE status and/or key predictors (*n* = 12). Therefore, 150 patients were included in the final analysis. Consequently, 150 patients were included in the final analysis. The mean age of participants was 48.67 ± 11.68 years, ranging from 18 to 83 years, with no significant age difference between the two groups (*p* = 0.28). These patients had significantly higher rates of neurovascular invasion and metastasis compared with those without PLE (*p* = 0.029 and *p* < 0.0001, respectively). Biomarker distributions (including ER and Ki‐67) are presented as reported in Table 1.

**Table 1:** Demographic and clinical characteristics of patients.

Characteristic	Total (150)	Lymphedema (+)	Lymphedema (‐)	*p*
Age (mean ± SD)	48.6 ± 11.7	50.8 ± 12.1	47.8 ± 11.2	0.28^a^
Metastasis				
No	120 (87.0%)	19 (15.8%)	101 (84.2%)	**< 0.0001** ^a^
Yes	18 (13.0%)	11 (61.1%)	7 (38.9%)	
Tumor location				
Upper left	61 (48.8%)	14 (23.0%)	47 (77.0%)	0.40^b^
Lower left	4 (3.2%)	0 (0.0%)	4 (100.0%)	
Upper right	56 (44.8%)	15 (26.8%)	41 (73.2%)	
Lower right	4 (3.2%)	2 (50.0%)	2 (50.0%)	
Tumor type				
Ductal	129 (91.5%)	28 (22.4%)	97 (77.6%)	1.000^a^
Lobular	12 (8.5%)	2 (18.2%)	9 (81.8%)	
Lymph node involvement				
No	67 (50.0%)	12 (18.2%)	54 (81.8%)	0.112^a^
Yes	67 (50.0%)	19 (30.2%)	44 (69.8%)	
Neurovascular invasion				
No	114 (85.1%)	23 (20.7%)	88 (79.3%)	**0.029** ^a^
Yes	20 (14.9%)	8 (44.4%)	10 (55.6%)	
ER positive				
No	64 (49.6%)	10 (15.9%)	53 (84.1%)	**0.032** ^a^
Yes	65 (50.4%)	20 (32.3%)	42 (67.7%)	
PR positive				
No	67 (51.9%)	13 (19.7%)	53 (80.3%)	0.233^a^
Yes	62 (48.1%)	17 (28.8%)	42 (71.2%)	
HER2 positive				
No	75 (58.1%)	18 (24.3%)	56 (75.7%)	0.919^a^
Yes	54 (41.9%)	12 (23.5%)	39 (76.5%)	
KI‐67 positive				
No	57 (44.2%)	7 (12.5%)	49 (87.5%)	**0.007** ^a^
Yes	72 (55.8%)	23 (33.3%)	46 (66.7%)	

^a^Pearson chi‐square.

^b^Fisher′s exact test.

### 3.2. Symptoms and Clinical Events

Patients who developed PLE reported significantly more swelling affecting daily activities and persistent swelling with skin discoloration than those without PLE (*p* < 0.0001 for both). “Swelling affecting daily activities” was defined as patient‐reported or clinician‐documented limitation in routine activities attributable to ipsilateral arm swelling. “Persistent swelling with skin discoloration” was defined as persistent ipsilateral swelling accompanied by documented skin color change (e.g., erythema or hyperpigmentation) not attributed to acute infection at that visit. Symptom frequencies and clinical events are summarized in Table 2.

**Table 2:** Symptoms and clinical events documented during follow‐up.

Symptom/event	Operational definition		Total (150) *N* (%)	Lymphedema (+)	Lymphedema (‐)	*p*
Swelling affecting daily activities	Patient‐reported or clinician‐documented functional limitation attributable to ipsilateral arm swelling	No	107 (73.8)	11 (10.3%)	96 (89.7%)	< 0.0001
		Yes	38 (26.2)	20 (52.6%)	18 (47.4%)	
Persistent swelling with skin discoloration	Persistent ipsilateral swelling with documented color change (e.g., erythema/hyperpigmentation) not attributed to acute infection at that visit	No	135 (93.1)	21 (15.6%)	114 (84.4%)	< 0.0001
		Yes	10 (6.9)	10 (100.0%)	0 (0.0%)	
Wound discharge	Any postoperative wound discharge documented after surgery	No	142 (97.9)	29 (20.4%)	113 (79.6%)	0.116
		Yes	3 (2.1)	2 (66.7%)	1 (33.3%)	
Post‐surgical trauma to affected arm	Documented trauma/injury to ipsilateral arm after surgery	No	144 (99.3)	30 (20.8%)	114 (79.2%)	0.214
		Yes	1 (0.7)	1 (100.0%)	0 (0.0%)	

### 3.3. Surgical and Treatment Factors

Surgical type was significantly associated with the development of PLE. Patients who underwent modified radical mastectomy (MRM) had higher rates of lymphedema (42.9%) compared with those who underwent breast‐conserving surgery (BCS) (16.0%) (*p* = 0.002). Additionally, patients who received adjuvant chemotherapy were more likely to develop PLE (*p* = 0.001). In contrast, radiotherapy did not show a significant correlation with the incidence of PLE (*p* = 0.259). The number of dissected lymph nodes was not significantly different between the groups (*p* = 0.465). These findings are summarized in Table 3.

**Table 3:** Surgical and treatment factors in lymphedema patients.

Characteristic	Total (150)	Lymphedema (+)	Lymphedema (‐)	*p*
Surgery type				
MRM	42 (34.1%)	18 (42.9%)	24 (57.1%)	**0.002** ^a^
BCS	81 (65.9%)	13 (16.0%)	68 (84.0%)	
Adjuvant chemotherapy				
No	34 (24.5%)	1 (2.9%)	33 (97.1%)	**0.001** ^b^
Yes	105 (75.5%)	30 (28.6%)	75 (71.4%)	
Radiotherapy				
Before	4 (4.2%)	2 (50.0%)	2 (50.0%)	0.259^b^
After	92 (95.8%)	22 (23.9%)	70 (76.1%)	
Dissected lymph nodes				
1–5	21 (16.5%)	2 (9.5%)	19 (90.5%)	0.373^a^
6–10	48 (37.8%)	13 (27.7%)	34 (72.3%)	
11–15	40 (31.5%)	11 (27.5%)	29 (72.5%)	
16–20	15 (11.8%)	3 (20.0%)	12 (80.0%)	
21–25	3 (2.4%)	1 (33.3%)	2 (66.7%)	

^a^Pearson chi‐square.

^b^Fisher′s exact test.

### 3.4. Logistic Regression Analysis

Logistic regression analysis identified metastasis as the most significant predictor of the development of stable lymphedema. The odds ratio for metastasis was 11.91 (95% CI: 1.94 to 73.09), indicating a strong association with the likelihood of developing lymphedema. The results of the logistic regression analysis are shown in Table 4.

**Table 4:** Logistic regression analysis for predictors of lymphedema.

Variable	B	Sig.	Exp (B)	95% CI for Exp (B)
Surgery type	−0.408	0.448	0.665	0.232–1.907
Adjuvant chemotherapy	1.694	0.118	5.439	0.650–45.549
Metastasis	2.477	**0.007** ^∗^	11.909	1.940–73.089
Neurovascular invasion	−0.027	0.972	0.973	0.208–4.548
ER positive	0.374	0.520	1.454	0.465–4.546
Ki‐67 positive	0.455	0.458	1.576	0.474–5.243

^∗^ *p* − value < 0.05 indicates statistical significance.

## 4. Discussion

This retrospective study evaluated clinical, surgical, and molecular correlates of PLE following modified ALND. Several factors—including surgery type, chemotherapy exposure, and tumor biomarkers (ER and Ki‐67)—were associated with PLE in univariable analyses. However, in the multivariable model, metastasis remained the only independent predictor, suggesting that other observed associations may be confounded by disease burden and treatment intensity.

First, metastasis was strongly associated with the development of PLE. Patients with metastasis exhibited a significantly higher risk of developing lymphedema. This finding is consistent with existing literature, which suggests that the spread of cancer cells to the axillary lymph nodes disrupts normal lymphatic function, impairing drainage and leading to the accumulation of lymph fluid, which results in edema [13]. Furthermore, the presence of metastasis increases the risk of lymphedema by causing fibrosis and obstructing lymphatic vessels, as described in earlier studies [14].

Mechanistically, metastatic involvement may reflect higher nodal disease burden and lymphatic obstruction, promoting lymphatic fibrosis and impaired drainage. In addition, systemic therapy—particularly taxane‐containing regimens—has been associated with endothelial inflammation and tissue remodeling, which may amplify susceptibility to clinically overt lymphedema when combined with surgical disruption of lymphatic pathways.

Next, the type of surgery performed was another significant risk factor for PLE. Patients who underwent MRM had a higher incidence of lymphedema compared with those who underwent BCS. This finding aligns with prior research indicating that more extensive surgeries, such as MRM, which involve the removal of multiple lymph nodes, increase the risk of lymphatic injury and subsequent development of lymphedema [15]. Our study did not find a significant difference in the number of lymph nodes dissected between groups, suggesting that other factors, such as the extent of lymph node involvement or surgical technique, may play a more important role in the development of lymphedema.

Chemotherapy emerged as another significant factor associated with the development of PLE. In our cohort, chemotherapy, particularly with taxanes, was strongly linked to the onset of PLE. Chemotherapy can exacerbate lymphatic dysfunction through mechanisms such as inflammation and peripheral neuropathy, which damage both the lymphatic vasculature and surrounding tissues, ultimately leading to fluid retention and edema [16, 17]. In support of this, Akezaki et al. demonstrated that breast cancer patients treated with neoadjuvant and adjuvant chemotherapy, specifically with docetaxel and cyclophosphamide, had an increased risk of developing lymphedema following ALND [18]. This underscores the importance of early identification and proactive management of lymphedema, particularly in patients receiving chemotherapy, and especially in those with additional risk factors.

When considering breast cancer, the status of ER, PR, HER2, and Ki‐67 markers, alongside lymph node involvement, plays a critical role in determining prognosis and guiding treatment decisions [19]. High Ki‐67 levels and positive lymph node status are generally associated with a higher risk of lymphedema development [20]; however, the exact relationship between these factors and lymphedema risk can vary depending on the specifics of each case. In our study, patients with ER‐negative and Ki‐67‐positive tumors were more likely to develop PLE. These molecular characteristics should be taken into account when assessing a patient′s risk for lymphedema, as they may help identify individuals who are at a higher risk of developing this challenging condition.

The observed PLE incidence (21.4%) lies within the wide range reported internationally for BCRL, which varies substantially by case definition, measurement approach, and follow‐up duration. Regarding tumor biology, ER‐negativity and high Ki‐67 are more prevalent in aggressive phenotypes (e.g., triple‐negative and other high‐proliferation subtypes) and may correlate with more intensive systemic therapy. In our study, these markers were associated with PLE in univariable analyses but did not remain independent predictors after adjustment, suggesting confounding by disease burden and treatment intensity.

### 4.1. Strengths

This study addresses a clinically important survivorship outcome after modified ALND and incorporates a spectrum of clinical, surgical, and tumor molecular variables (ER/PR/HER2/Ki‐67) that are not consistently available in all lymphedema datasets.

### 4.2. Limitations

First, the retrospective single‐center design limits causal inference and may reflect local practice patterns. Second, selection bias and limited external validity are possible because we excluded patients with factors that markedly alter baseline lymphedema risk or outcome ascertainment (e.g., BMI ≥ 35 kg/m^2^, advanced stage, neoadjuvant radiotherapy, postoperative infection, prior upper‐limb/neck trauma, and prior treatment at other institutions); therefore, the incidence and risk estimates may not generalize to higher‐risk populations and may underestimate the overall burden of PLE. Third, outcome ascertainment/measurement bias may be present because PLE classification relied on documentation in medical records and objective standardized limb measurements were not uniformly available across all patients, which may result in misclassification. Fourth, missingness and attrition bias are possible because some patients were excluded because of mortality, noncooperation, or incomplete records; excluded patients may have differed systematically from included participants, and the mechanism of missingness could not be fully characterized. Fifth, although we considered clinically plausible predictors, residual confounding cannot be excluded, and multivariable adjustment was necessarily constrained by the limited number of PLE events (*n* = 31), increasing the risk of model instability and restricting the number of covariates that could be entered without overfitting; accordingly, adjusted estimates—particularly for predictors not retained as significant—should be interpreted cautiously. Prospective multicenter studies with standardized objective lymphedema assessment and longer follow‐up are warranted.

## 5. Conclusion

In conclusion, although surgery type, chemotherapy exposure, and tumor biomarkers (ER and Ki‐67) were associated with PLE in unadjusted analyses, metastasis was the only independent predictor in multivariable regression. Clinically, patients with metastatic involvement may warrant intensified surveillance and early referral for lymphedema assessment. Prospective multicenter studies with standardized objective limb measurement and adequate sample size are required to confirm independent predictors.

## Author Contributions

**Iraj Feizi:** conceptualization, methodology, formal analysis, supervision, writing – review and editing. **Bahareh Nikandam:** data curation, investigation, visualization, writing – review and editing. **Zohreh Shahabi:** software, formal analysis, project administration, validation, writing – review and editing. **Amir Ahmad Arabzadeh:** conceptualization, methodology, writing – original draft, writing – review and editing.

## Funding

No funding was received for this manuscript.

## Conflicts of Interest

The authors declare no conflicts of interest.

## Data Availability

The data that support the findings of this study are available from the corresponding author upon reasonable request.
